# Determination of Theophylline Across Biological, Environmental and Food Matrices Using Liquid-Phase Microextraction Coupled with LC-MS/MS

**DOI:** 10.3390/molecules30183797

**Published:** 2025-09-18

**Authors:** Bin Lin, Fen Wang, Hongliang Wang, Xinsheng Huang, Xueqin Liu, Xuechun Wang, Chihua Wang, Yan Xing, Chunqing Dai, Yi Zheng

**Affiliations:** 1 Key Laboratory of Food Safety Risk Monitoring for Health Commission of Hubei Province, Huanggang Municipal Center for Disease Control and Prevention, Huanggang 438000, China; linbin0722@126.com (B.L.);; 2 Fujian Center for Drug Inspection of Fujian Medical Products Administration, Fuzhou 350003, China

**Keywords:** theophylline, sample preparation, liquid-phase microextraction, LC-MS/MS, multi-matrix analysis

## Abstract

Theophylline represents a significant public health challenge due to its dual acute and chronic toxicity resulting from therapeutic, environmental, and dietary exposures. Effective monitoring across the bio-environmental–food triad requires analytical methods that are highly sensitive, universally applicable, and capable of overcoming complex matrix interferences. This study introduces a flat membrane-based liquid-phase microextraction (LPME) technique combined with LC–MS/MS for the determination of theophylline in diverse matrices. The method eliminates the need for specialized adsorbents or equipment, offering a simple and cost-effective solution for high-throughput sample clean-up. Under optimized conditions, the method demonstrated exceptional sensitivity (LOD: 0.2 ng mL^−1^) and a wide linear range (0.01–10 μg mL^−1^). It was successfully applied to plasma, urine, hospital sewage, and green tea, providing accurate (recoveries of 86.7–111.3%) and reproducible (RSD < 10%) results across all matrices. This unified and robust approach effectively addresses matrix interferences and provides a reliable tool for the monitoring and risk assessment of theophylline across multiple domains.

## 1. Introduction

Theophylline has long been a cornerstone bronchodilator for respiratory diseases such as asthma and COPD [1]. Nevertheless, its therapeutic use is complicated by dose-dependent dual toxicity and a narrow therapeutic window (10–20 μg mL^−1^) [2]. The drug is predominantly metabolized by hepatic cytochrome P450 1A2 (CYP1A2), exhibiting substantial interindividual variability due to genetic polymorphisms, drug interactions (e.g., with fluoroquinolones or macrolides), lifestyle factors (e.g., smoking), and hepatic function [2,3]. Its pharmacodynamic actions involve non-selective adenosine receptor antagonism and phosphodiesterase inhibition, which mediate both its bronchodilatory effects and dose-dependent toxicities [3,4]. Acute neurocardiac toxicity—manifesting as seizures and tachyarrhythmias—occurs at supratherapeutic concentrations (>20 μg mL^−1^) and necessitates rigorous therapeutic drug monitoring (TDM) [5]. Actually, TDM is indicated not only at therapy initiation but also during disease exacerbations, upon suspicion of toxicity, or when alterations in pharmacokinetics occur [6]. In addition, chronic low-dose exposure via non-clinical routes—including environmental sources (e.g., aquatic pseudo-persistence at concentrations ranging from hundreds of ng L^−1^ to several µg L^−1^) [7]), dietary intake (beverages and botanicals containing 0.05–50 μg mL^−1^) [8], and metabolic variations (e.g., 1.65-fold plasma accumulation in CYP1A2-deficient elderly patients) [9]—can lead to insidious multi-organ damage independently of acute toxicity.

Critically, environmental and dietary sources interact synergistically with biological factors, amplifying population-scale cardioneurological injury [10,11]. While traditional clinical TDM remains essential for managing acute toxicity, its blood-centric focus is inadequate for intercepting chronic, multi-source exposure [12]. Therefore, effective public health protection requires a comprehensive surveillance technique encompassing the bio-environmental–food triad [13]. Such an integrated system combines blood-based TDM with monitoring of human biospecimens, aquatic systems, and food matrices. This holistic approach is vital to map exposure trajectories, quantify cumulative risk, and pre-empt toxicity across all pathways [12].

Current quantification of theophylline primarily employs techniques such as ultraviolet-visible spectroscopy (UV-Vis) [14], immunoassay [15], high-performance liquid chromatography (HPLC) [16], and liquid chromatography–tandem mass spectrometry (LC-MS/MS) [17,18]. Among these, LC–MS/MS is considered the gold standard due to its exceptional sensitivity, selectivity, and throughput [19]. However, all these methods face significant matrix-derived challenges across biological, environmental, and food samples [20,21]. For instance, UV-Vis lacks the sensitivity required for trace detection in complex matrices [22]; immunoassays are limited by structural cross-reactivity [23]; HPLC is susceptible to endogenous co-elution in biological specimens [24]; and LC-MS/MS experiences matrix-specific ion suppression or enhancement in complex samples (e.g., due to albumin/salts in biospecimens [25,26], humic acid in environmental waters [27], or tannins in food extracts [28]). Thus, robust extraction techniques are essential prior to instrumental analysis for reliable surveillance across the bio-environmental–food triad.

To mitigate matrix interference, researchers have developed methods using tailored adsorbent materials [29,30,31]—such as copper-doped magnetic microspheres for plasma and molecularly imprinted polymers for serum—or phase-separation approaches like solvent-emulsification microextraction [32]. Electromembrane extraction has also shown efficacy for plasma and urine matrices [33]. However, these methods face two major limitations: (i) validation is typically confined to specific specimen types, lacking cross-domain applicability; and (ii) large-scale monitoring requires high-throughput processing, but material synthesis and specialized equipment increase costs and operational complexity. Therefore, there is an urgent need for a universally applicable, cost-effective extraction technique compatible with high-throughput demands.

Membrane-based liquid-phase microextraction (LPME), an evolution of supported liquid microextraction, offers a promising solution due to its exceptional purification and preconcentration capabilities [34,35,36,37,38]. In LPME, passive transport of neutral analytes across a supported liquid membrane (SLM) is driven by a concentration gradient, followed by electrostatic trapping in the acceptor phase via charge-mediated partitioning [39]. Among LPME configurations, flat membrane-based LPME (FM-LPME) stands out due to its operational flexibility and inherent compatibility with automation, as demonstrated in our previous works using wide-end sealed pipette tips [40,41]. This miniaturized design meets the bio-environmental–food triad: streamlined parallel processing enables scalable sample handling; physical isolation prevents cross-contamination; and membrane-based purification mitigates matrix effects without costly materials.

**Figure 1:**
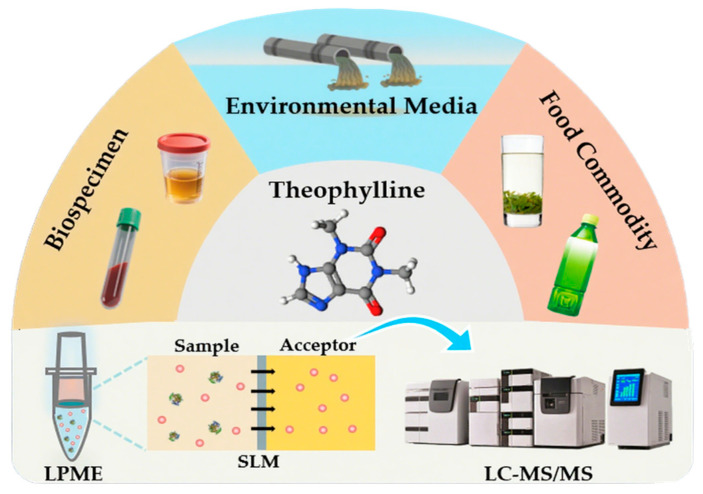
Schematic illustration of the proposed LPME-LC-MS/MS system.

In this study, we present the first integrated LPME-LC-MS/MS method for the determination of theophylline in biological (plasma, urine), environmental (hospital sewage), and food (green tea) matrices (Figure 1). The platform synergizes miniaturized FM-LPME—which enables universal matrix purification by eliminating domain-specific interferences—with ultrahigh-performance LC–MS/MS, providing exceptional sensitivity and selectivity for trace-level quantification. This unified workflow establishes a robust foundation for future comprehensive exposure mapping and cumulative risk assessment within bio-environmental–food triad surveillance.

## 2. Results and Discussion

### 2.1. Optimization of Extraction Parameters

To optimize extraction of theophylline from multiple matrices, key factors—including organic solvent type, solvent volume, donor and acceptor phase composition, extraction temperature, and extraction time—were systematically investigated. In this work, the extraction recovery representing separation efficiency of theophylline is defined by the following equation:

$$Recovery\left(\%\right)=C_{A}\left(t\right)\times \frac{V_{A}}{C_{D}^{0}{\times V}_{D}}\times 100\%$$

where $C_{D}^{0}$ is the initial concentration of theophylline in the donor phase, $C_{A}\left(t\right)$ is the time-dependent concentration of theophylline in the acceptor phase, and $V_{D}$ and $V_{A}$ are the volume of the donor and acceptor phases, respectively.

#### 2.1.1. Selection of Organic Solvent

**Figure 2:**
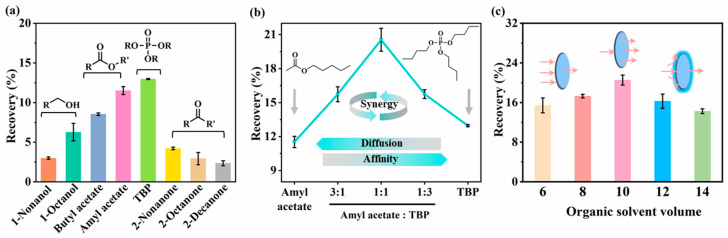
(**a**) Effect of organic solvents on theophylline recovery. (**b**) Effect of amyl acetate/TBP volume ratio on theophylline recovery. (**c**) Effect of organic solvent volume on theophylline recovery. Extraction conditions: donor phase: 10 μM HCl (pH = 5); acceptor phase: 10 mM NaOH (pH = 12); extraction time: 30 min; temperature: 40 °C. Solvent volume for (**a**,**b**): 10 μL; solvent for (**c**): TBP/amyl acetate (1:1, *v*/*v*).

The choice of organic solvent critically influences LPME efficiency, requiring high stability, low aqueous solubility, and strong analyte affinity [42]. Solvents tested included 1-octanol, 1-nonanol, butyl acetate, amyl acetate, tributyl phosphate (TBP), 2-octanone, 2-nonanone, 2-decanone. TBP yielded the highest theophylline recovery (Figure 2a), likely due to its polarized P=O group potentially forming stronger hydrogen bonds with theophylline’s N-H group [43]. However, its high viscosity limits analyte diffusivity [44,45]. Amyl acetate, the second-best performer, offers lower viscosity and faster diffusion. Blending TBP with amyl acetate at varying ratios improved extraction efficiency, with a 1:1 (*v*/*v*) ratio achieving optimal recovery (Figure 2b). This combination balances high affinity with improved diffusion [46]. Thus, TBP/amyl acetate (1:1, *v*/*v*) was selected for the subsequent experiments.

Solvent volume also significantly affected recovery (Figure 2c). Recovery increased from 6 μL to 10 μL due to enhanced interfacial contact area and improved analyte transfer [47]. Beyond 10 μL, recovery decreased, likely due to an elongated diffusion path and impaired mass transfer kinetics [48]. Therefore, 10 μL was chosen as the optimal volume.

#### 2.1.2. Optimization of Donor Phase

**Figure 3:**
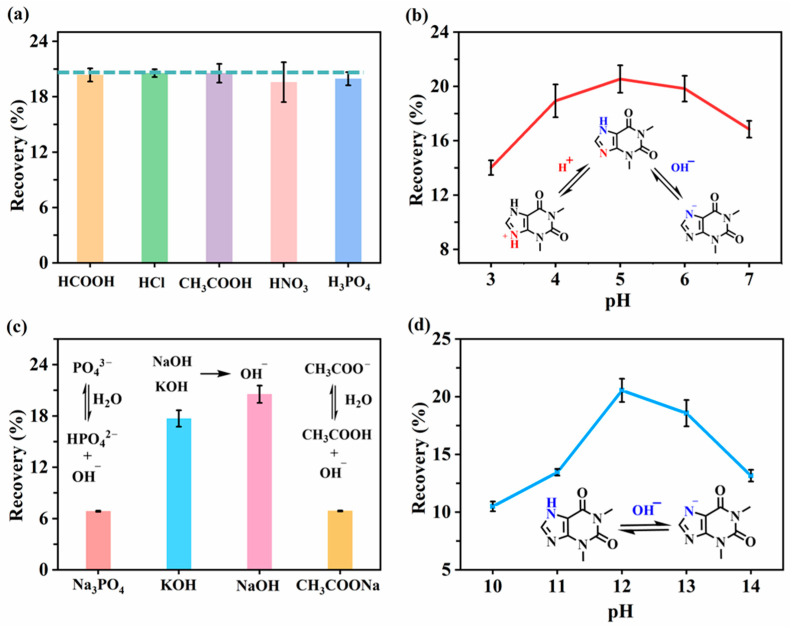
(**a**) Effect of different acids in donor phase on theophylline recovery. (**b**) Effect of donor phase pH on theophylline recovery. (**c**) Effect of different alkalis in acceptor phase on theophylline recovery. (**d**) Effect of acceptor phase pH on theophylline recovery. Extraction conditions: organic solvent: 10 μL TBP/amyl acetate (1:1, *v*/*v*); extraction time: 30 min; temperature: 40 °C.

Based on LPME principles, efficient SLM transfer requires the analyte to be in its neutral form [49]. Theophylline (pKa = 8.81) can exist as cationic, neutral, or anionic species depending on pH [50,51,52,53,54,55]. To maximize the neutral fraction, the donor phase was acidified. Several acids including formic acid, hydrochloric acid, acetic acid, nitric acid, phosphoric acid at 10 μM concentration were first evaluated. As shown in Figure 3a, no significant difference in extraction recovery was observed across the acids. This indicated that pH, rather than the anion type, was the dominant factor [47]. Given its prevalence as the LPME electrolyte [40], HCl was selected for subsequent experiments. Then, the effect of donor phase pH on the recovery was investigated. Extraction efficiencies exhibited a pH-dependent trajectory (Figure 3b). Recovery was highest at pH 5 (Figure 3b), where theophylline is predominantly neutral [53]. Higher pH values led to deprotonation and reduced extraction efficiency [54]. Thus, 10 μM HCl (pH = 5) was chosen as the donor phase.

#### 2.1.3. Optimization of Acceptor Phase

Consistent with LPME principles, effective trapping requires ionization of analyte in the acceptor phase to prevent back-diffusion [56]. Four alkaline solutions (sodium phosphate, potassium hydroxide, sodium hydroxide, and sodium acetate) at 10 mM concentration were evaluated. Depicted in Figure 3c, hydroxide yielded the highest recovery, likely due to its strong alkalinity and favorable ion kinetics. Subsequent pH optimization (range: 10–14) showed peak efficiency at pH 12 (Figure 3d), indicating near-complete ionization. Beyond pH 12, efficiency declined sharply, potentially due to analyte decomposition under highly alkaline conditions [57]. Consequently, 10 mM NaOH (pH = 12) was selected as acceptor phase.

#### 2.1.4. Optimization of Temperature and Time

**Figure 4:**
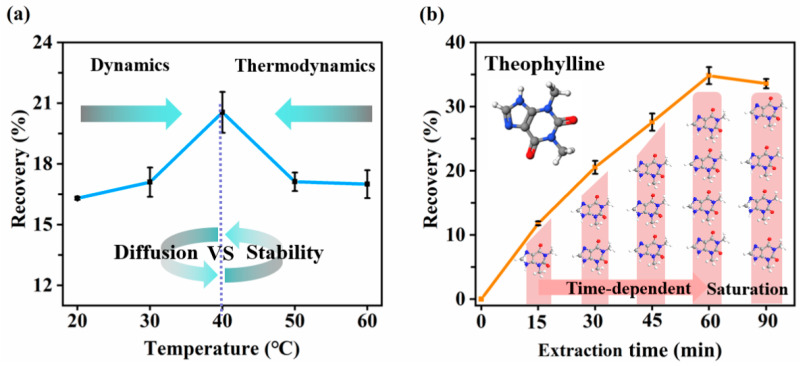
(**a**) Effect of extraction temperature on theophylline recovery. (**b**) Effect of extraction time on theophylline recovery. Extraction conditions: organic solvent: 10 μL TBP/amyl acetate (1:1, *v*/*v*); donor phase: 10 μM HCl (pH = 5); acceptor phase: 10 mM NaOH (pH = 12). Extraction time for (**a**) was 30 min and temperature for (**b**) was 40 °C.

Following comprehensive optimization of organic solvent composition and phase conditions, extraction temperature and time were systematically evaluated to maximize theophylline recovery. Temperature critically modulates mass transfer kinetics by influencing molecular diffusivity and membrane solvent properties [49]. As shown in Figure 4a, recovery increased with temperature up to 40 °C, aided by the dominant effect of enhanced diffusivity. Beyond 40 °C, solvent evaporation reduced SLM stability and recovery [58]. Subsequently, extraction time optimization revealed a dynamic kinetic profile (Figure 4b). Hence, an extraction temperature of 40 °C was selected in this study. Subsequently, the effect of extraction time on theophylline extraction was investigated. Depicted in Figure 4b, recovery progressively increased with time, reaching equilibrium at 60 min. Prolonging the extraction time showed no recovery improvement, indicating mass transfer saturation [47]. Therefore, 60 min was chosen as the optimal extraction time.

### 2.2. Validation of Analytical Method

**Figure 5:**
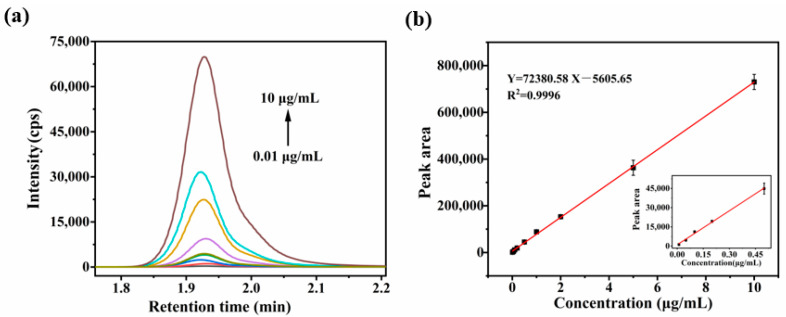
(**a**) Total ion chromatograms of theophylline at different concentrations. (**b**) Calibration curve of the LPME-LC-MS/MS method.

To evaluate the analytical performance of proposed LPME-LC-MS/MS method, the figures of merit including linear range, limit of detection (LOD) and limit of quantification (LOQ) were systematically investigated. The total ion chromatogram and calibration curve were formed for theophylline analysis at a series of concentrations. As shown in Figure 5a, the total ion chromatogram showed a concentration-dependent increase in signal intensity. This enabled the establishment of the calibration curve (Figure 5b), which demonstrated excellent linearity (correlation coefficient > 0.999) over the concentration range of 0.01–10 μg mL^−1^. Furthermore, the LOD and LOQ, determined based on signal-to-noise ratios (S/N) of 3 and 10, were found to be 0.2 ng mL^−1^ and 0.7 ng mL^−1^, respectively.

### 2.3. Analysis of Real Samples

The applicability and universality of the developed method were rigorously validated by applying it to four strategically selected matrices that represent the major exposure routes within the bio-environmental–food triad: plasma (therapeutic monitoring), urine (excretion biomarker), hospital sewage (environmental discharge), and green tea (dietary intake). This choice was designed to challenge the method with a wide range of domain-specific interferents. No endogenous theophylline was detected in plasma and urine, whereas sewage and tea contained 0.09 μg mL^−1^ and 0.18 μg mL^−1^, respectively. Subsequently, spiking studies were performed to test the accuracy and precision of this method. As detailed in Table 1, the method demonstrated excellent accuracy and precision across all tested concentration levels within each matrix. For each concentration level (*n* = 3), the mean recoveries fell within the accepted range of 80–120%, and the associated 95% confidence interval (CI) also resided within these acceptable bounds. The precision, expressed as relative standard deviation (RSD), was consistently below 10% at all levels, confirming the method’s robustness throughout its calibrated range.

### 2.4. Comparison with Other Methods

Table 2 provides a critical comparison between the proposed LPME-LC-MS/MS method and established extraction-based techniques for the analysis of theophylline, highlighting its superior performance across key metrics. Notably, the present method achieves significantly enhanced sensitivity (LOD = 0.2 ng mL^−1^) and a wider linear dynamic range (0.01–10 μg mL^−1^), attributable to the synergistic combination of optimized LPME preconcentration and the high selectivity of LC-MS/MS. This performance enables reliable quantification spanning trace environmental levels to therapeutic concentrations. Operationally, the integrated LPME approach offers distinct advantages: it requires no synthesis of specialized materials (e.g., molecularly imprinted polymers) [29] or additional external equipment (e.g., electrodes as used in electromembrane extraction) [33], while the supported liquid membrane (SLM) inherently cleans up samples by physically isolating the analyte from complex matrices. Crucially, the method demonstrates broad applicability across biological (plasma, urine), environmental (sewage), and food (tea) matrices without requiring protocol modifications. Overall, current LPME-LC-MS/MS platform represents an optimal balance of high sensitivity, wide linearity, operational simplicity, inherent matrix isolation capability, and cross-matrix versatility, making it highly suitable for the determination of theophylline in real-world samples.

## 3. Materials and Methods

### 3.1. Chemicals and Materials

Theophylline was obtained from Shanghai Maclean Biochemical Technology Co., Ltd. (Shanghai, China). 1-nonanol, 1-octanol, butyl acetate, amyl acetate, TBP, 2-nonanone, 2-octanone and 2-decanone were purchased from Shanghai Aladdin Biochemical Technology Co., Ltd. (Shanghai, China). Formic acid, hydrochloric acid, acetic acid, nitric acid, sulfuric acid, sodium phosphate, potassium hydroxide, sodium hydroxide and sodium acetate were supplied by Sinopharm Chemical Reagent Co., Ltd. (Shanghai, China). Methanol was purchased from Thermo Fisher (Tianjin, China). Formic acid, methanol and acetonitrile were of chromatographic purity grade while other chemicals were of analytical grade. Milli-Q water purification system from Merck (Darmstadt, Germany) was employed to produce deionized water. Plasma samples were purchased from Northern Weiye Metrology Group Co., Ltd. (Xinyang, China). Urine samples were collected from healthy volunteers. Hospital sewages were collected from a general hospital in Huanggang city. Green tea samples were obtained from Inner Mongolia Yili Industrial Group Co., Ltd. (Hohhot, China). Accurel polypropylene 2E (R/P) membrane with a thickness of 200 μm was supplied by Membrana (Wuppertal, Germany). The 1000 μL pipette tips were purchased from Kirgen (Shanghai, China), while 2 mL polypropylene tubes were obtained from Bkman (Changde, China).

### 3.2. Extraction Setup and Procedures

As described in our previous works [40,41], the FM-LPME setup comprised two major components including a 2 mL Eppendorf PP tube and a wide end-closed pipet tip with a piece of flat membrane, which served as the donor phase compartment and acceptor phase compartment, respectively. For LPME extraction, the organic solvent was first immobilized on the thin polypropylene flat membrane to form the SLM. Then, the analyte was extracted from 1000 μL of donor phase across the SLM and further transferred into 100 μL of acceptor phase. The setup was agitated by an MIC-100 mixer (Hangzhou MIULAB Instrument Co., Ltd., Hangzhou, China) at a speed of 1500 rpm to initiate the extraction process. Upon completion of the preset extraction time, the extraction was automatically terminated. Subsequently, the acceptor solution after extraction was immediately collected and analyzed by LC-MS/MS.

### 3.3. Method Validation and Application

To assess the quantitative characteristics of proposed method, extractions were performed under the optimal conditions with theophylline at concentrations of 0.01, 0.05, 0.1, 0.2, 0.5, 1, 2, 5 and 10 μg mL^−1^ (*n* = 5 for each concentration). The acceptor solution after LPME was then analyzed by LC-MS/MS. The peak area of theophylline versus analyte concentration was plotted to construct the calibration curve. In the processing of collected samples with different matrices, all original samples were first adjusted to the pH of 5 with HCl solution. For analysis of blank samples, hospital sewage, urine, green tea and plasm samples were diluted two, five, eight and ten times with 10 μM HCl prior to extraction. For the spiked experiments, the desired amount of theophylline was directly added into the untreated samples, followed by the same pH adjustment and HCl dilution as described in blank sample analysis.

### 3.4. LC-MS/MS Analysis

Theophylline analysis was implemented using a 1290 Infinity II ultra-performance liquid chromatography (UPLC) system equipped with a 6475 triple quadrupole Mass Spectrometry (Agilent, Santa Clara, CA, USA). The column was a Zorbax Eclipse Plus C18 column (2.1 mm × 50 mm, 1.8 μm) from Agilent (Santa Clara, CA, USA) with gradient elution, and was maintained at 40 °C. The mobile phase A consisted of MQ-water containing 2 mM ammonium acetate and 0.1% formic acid (*v*/*v*), while mobile phase B was acetonitrile. Formic acid was added to maintain peak shapes. The flow was kept at 0.4 mL min^−1^ and the injection volume was 1 μL. The elution process was performed according to the following procedures: mobile phase B started from 5% for 1.5 min and elevated to 95% in 1.5 min; subsequently, 95% mobile phase B was kept for 2 min; ultimately, mobile phase B was decreased to 5% within 0.1 min. Detection was conducted with an electrospray ionization source in a negative-ionization mode. The sheath gas was 11 Arb, while the aux gas was 8 Arb. The capillary temperature was 350 °C and the vaporizer temperature was 300 °C. The spray voltage was set at 4.0 kV. Target identification and quantification were performed using multiple reaction monitoring. The monitored ion transitions were *m*/*z* 181.1 → 96.0 and *m*/*z* 181.1 → 124.1. The fragmentor voltages were 110 V, while the collision energies consisted of 25 eV and 18 eV.

## 4. Conclusions

In conclusion, this study addresses a pivotal technical challenge within the emerging paradigm of integrated bio-environmental–food exposure assessment. We have successfully developed and validated a unified, matrix-agnostic analytical platform for theophylline based on LPME-LC-MS/MS. The method’s high sensitivity, robustness, and demonstrated applicability across biological, environmental, and dietary matrices directly translate into a practical and powerful tool for future surveillance initiatives. By enabling the accurate quantification of theophylline from multiple exposure sources using a single protocol, this work provides the much-needed analytical foundation to map population exposure trajectories, quantify cumulative risk, and ultimately bridge the gap between traditional clinical TDM and comprehensive public health protection. The proposed strategy could serve as a blueprint for monitoring other emerging contaminants of concern within a one-health context.

## Figures and Tables

**Table 1:** Accuracy and precision of the proposed method in different matrices.

Matrix	Background (μg mL^−1^)	Spiked (μg mL^−1^)	Detected (μg mL^−1^)	Accuracy (%)	95% CI	RSD (%)
Plasma	ND	0.1	0.09	91.1	77.1–105.0	6.18
		0.2	0.17	87.2	74.3–100.1	6.01
		0.5	0.43	86.7	68.8–104.6	8.57
Urine	ND	0.1	0.11	107.9	105.2–110.5	1.04
		0.2	0.19	96.3	91.9–100.7	1.77
		0.5	0.48	95.3	89.8–100.7	2.25
Hospital sewage	0.09	0.2	0.30	103.3	87.2–119.3	6.20
		1	1.15	105.4	92.1–118.7	5.06
		5	5.56	109.3	104.5–114.1	1.81
Green tea	0.18	0.2	0.39	103.8	98.9–108.7	1.94
		1	1.18	99.1	96.9–101.3	0.83
		5	5.75	111.3	105.5–117.1	2.10

ND indicated no theophylline residual was detected.

**Table 2:** Comparison of the proposed method with other extraction-based techniques for theophylline determination.

Analytical Method	Extraction Principle	Sample	LOD (μg L^−1^)	Linear Range (μg mL^−1^)	Reference
MIP ^a^-HPLC-UV	Material adsorption	Green tea	10	0.1–100	[29]
MSPE ^b^-HPLC-UV	Material adsorption	Plasma	3	0.02–20	[30]
MIP ^a^-SPE-HPLC-UV	Material adsorption	Serum	90	0.5–30	[31]
SEME ^c^-HPLC-UV	Emulsification transfer	Cocoa powder/Plasma	0.15	0.002–0.15	[32]
EME ^d^-HPLC-UV	Electric migration	Plasma/Urine	15	0.05–0.5	[33]
LPME-LC-MS/MS	Passive diffusion	Plasma/Urine/Hospital sewage/Green tea	0.2	0.01–10	This work

^a^ Molecularly imprinted polymer. ^b^ Magnetic solid-phase extraction. ^c^ Surfactant-enhanced emulsification microextraction. ^d^ Electromembrane extraction.

## Data Availability

No new data were generated in this study. Data sharing is not applicable to this article.

## Abbreviations

The following abbreviations are used in this manuscript:

COPD	Chronic obstructive pulmonary disease
CYP1A2	Cytochrome P450 family 1 subfamily A member 2
TDM	Therapeutic drug monitoring
UV-Vis	Ultraviolet–visible spectroscopy
HPLC	High-performance liquid chromatography
LC-MS/MS	Liquid chromatography–tandem mass spectrometry
LPME	Liquid-phase microextraction
SLM	Supported liquid membrane
FM-LPME	Flat membrane-based LPME
TBP	Tributyl phosphate
LOD	Limit of detection
LOQ	Limit of quantification
Cl	Confidence interval
RSD	Relative standard deviation
UPLC	Ultra-performance liquid chromatography
